# Investigating Acupoint Selection and Combinations of Acupuncture for Tic Disorders: An Association Rule Mining and Network Analysis Study

**DOI:** 10.3389/fneur.2022.894951

**Published:** 2022-06-10

**Authors:** Jieting Chen, Yufeng Xie, Qingchan Lin, Ziliang Qian, Jun Feng, Jianmei Zhang, Yun Chen, Wenhan Chen, Yueting Wu, Ziyi Guo

**Affiliations:** ^1^Shenzhen Hospital of Guangzhou University of Chinese Medicine, Shenzhen, China; ^2^Sixth Clinical School of Medicine, Guangzhou University of Chinese Medicine, Shenzhen, China; ^3^Macau University of Science and Technology, Taipa, Macao SAR, China

**Keywords:** acupuncture, tic disorders acupoint, data mining, association rule, cluster analysis

## Abstract

**Objective:**

Tic disorders (TDs) are common mental disorders in children and adolescents, and the clinical application of acupuncture for treating TDs is becoming increasingly widespread. However, the criteria for selecting acupoint prescriptions and combinations have not been summarized. Therefore, data mining was used herein to determine the treatment principles and the most effective acupoint selection and compatibility criteria for the treatment of TDs.

**Methods:**

Clinical studies and observations of the efficacy of acupuncture treatment for TDs were obtained from the PubMed, Cochrane Library, EMBASE, China National Knowledge Infrastructure (CNKI), Wanfang, VIP, and Chinese Biomedical (CBM) databases. The data on the acupoint prescriptions applied in these studies were collected, and network and association analyses were used to reveal the relationships between acupoints and to identify acupoint combinations. Additionally, the principles of acupuncture for TDs were determined through cluster analysis. Subgroup analysis of acupuncture prescriptions based on specific categorical diagnoses was performed to further assess the selection of acupoints.

**Results:**

Eighty-six trials were identified, and 257 groups of effective prescriptions involving 121 acupoints were extracted. Bai-hui (DU20), Feng-chi (GB20), Tai-chong (LR3), He-gu (LI4), and San-yin-jiao (SP6) were the most regularly used acupoints for treating TDs. The Governor Vessel, gallbladder, and large intestine meridians were more commonly used than other meridians. Moreover, most acupoint sites focused on the head and neck. Network analysis revealed potentially effective acupoint prescriptions for their commonly used acupoints, namely, Bai-hui (DU20), Si-shen-cong (EX-HN1), Feng-chi (GB20), Nei-guan (PC6), Shen-men (HT7), He-gu (LI4), Zu-san-li (ST36), San-yin-jiao (SP6) and Tai-chong (LR3). Association rule mining indicated that potential point combinations that should be prioritized in TD treatment are Bai-hui (DU20), Neiguan (PC6) and Sanyinjiao (SP6). Cluster analysis revealed the treatment principle of “coordinating yin and yang, tonifying qi and blood, dispelling pathogenic wind and eliminating phlegm”. The core acupoint prescription of TS treatment comprised He-gu (LI4), Feng-chi (GB20), Tai-chong (LR3), Bai-hui (DU20), Yin-tang (EX-HN3), Si-shen-cong (EX-HN1), San-yin-jiao (SP6), and Nei-guan (PC6). The core group included He-gu (LI4) and Feng-chi (GB20). Proximal points were usually used in TS as an additional method of point selection.

**Conclusion:**

Using data mining analysis of published studies, this study provides valuable information regarding the selection of the most effective acupoints and point combinations for clinical acupuncture practice for treating TDs.

## Introduction

A tic disorder (TD) is a neuropsychiatric disorder that occurs mostly in childhood (1). The clinical manifestations of TDs are several motor and at least one phonic tic, which may be accompanied by attention deficit hyperactivity disorder, sleep disorders, mood disorders and other neuropsychiatric disorders (2). The pathogenic mechanisms of TDs are currently unknown. Several studies have shown that TDs are mainly associated with neurotransmitter imbalance, whiplash brain injury, and imbalance in micronutrient intake (1). Currently, typical antipsychotics, atypical antipsychotics, painkillers and other medications are commonly used as treatments in clinical practice. Unfortunately, these medicines cannot cure TD and are accompanied by adverse effects such as drowsiness and weight gain. Other main treatments are neuromodulation therapy and psychobehavioral therapy (3). TD is characterized by tics as the main symptom and are characterized as disorders of the Endogenous Liver Wind in traditional Chinese medicine (TCM). The pathogenesis of TD is mainly due to the deficiency of the five zang organs, the imbalance of yin and yang, and the interlocking of Wind-phlegm. Previous research has shown that TCM therapies such as acupuncture, massage and Chinese herbal treatment can be used to treat TDs (4–7). Over the years, the application of acupuncture in the treatment of TDs has become increasingly widespread and has achieved good efficacy (8). We used data mining techniques to analyze and explore the regularity of clinical acupuncture point selection for treating TDs to provide a reference for the standard treatment of TDs with acupuncture.

## Materials and Methods

### Literature Source and Search Strategy

The electronic literature databases PubMed, the Cochrane Library, EMBASE, China National Knowledge Infrastructure (CNKI), Wanfang, VIP, and Chinese Biomedical (CBM) were searched for research on acupuncture treatments for TDs published from the inception of the database to 10th October 2021, with the language restricted to English and Chinese. The search strategy used subject terms related to acupuncture and TDs, such as convulsion disorder, acupuncture, and electroacupuncture.

### Literature Screening

Two researchers independently screened the eligible articles according to the inclusion and exclusion criteria; if these two researchers disagreed about an article, the article was screened by a third researcher. After reading the titles and abstracts, we deleted duplicate and irrelevant articles. Then, according to the inclusion and exclusion criteria, the remaining articles were further screened.

#### Inclusion Criteria

The inclusion criteria were as follows:

- Randomized controlled trials (RCTs) and self-control and retrospective studies (NRSIs) were included.
- The participants described in the studies met one of the following diagnostic criteria for TDs: American Handbook of Diagnostic Statistics of Mental Disorders, Chinese Classification Scheme and Diagnostic Criteria of Mental Disorders, Practical TCM Pediatrics, TCM Pediatrics, Chinese Society of Traditional Chinese Medicine Guidelines for the Treatment of Common Pediatric Disorders, Neurology. The age of the participants was <20 years old, with no restrictions on sex, race, or duration of illness.
- There were recognized evaluation criteria for efficacy, and the therapeutic group efficacy was clearly reported. Acupuncture therapy was defined as manual acupuncture or electroacupuncture points, moxibustion, acupuncture and moxibustion with simultaneous intervention, massage, cupping, acupoint injection, acupoint needle embedding, acupoint catgut embedding, and a combination of two or more of these therapies. In addition, a clear acupoint prescription that matched the acupoint trigger points was reported.
- The primary outcome indicators were recognized efficacy evaluation criteria, such as the Yale gestalt gross severity scale (YGTSS) score, open illness scoring method score, and quantitative TCM evidence grading score. The efficacy of the intervention group was clearly reported.
- Republished articles retained only the most recent article.

#### Exclusion Criteria

The exclusion criteria were as follows:

- Newspapers, conference papers, degree papers, research on mechanisms, animal experiments, systematic reviews or meta-analyses, case reports and theory categories were excluded.
- Studies with ambiguous acupoint prescriptions, inability to extract clear acupoint prescriptions that matched the acupoint trigger points, and no significant efficacy in the intervention group were excluded.
- The study records were not clearly reported, and the evaluation criteria were not standardized.

### Data Extraction and Quality Assessment

One reviewer extracted informational data for all the eligible studies, including the name of the first author, time of issue, title of the article, type of study, sample size, diagnostic criteria, age of the participants, interventions, principle acupoints and additional acupoints, and outcome measures. Valid prescriptions were extracted *via* the “one group of main acupoints and one group of minor acupoints with one group of acupoint prescriptions” strategy. Another reviewer checked for the accuracy and completeness of the input information. The names of all the acupoints included in the prescription were standardized according to the World Health Organization's Standard Acupuncture Point Location in the Western Pacific Region and the China National Standard “Naming and Positioning of Acupoints” (GB/T 12346-2006). The meridians, positioning of acupoints and special points were extracted. Finally, we established a dataset of acupuncture treatment for TDs and entered all the data into a Microsoft Excel 2019 workbook.

Using the revised Cochrane risk-of-bias tool for randomized trials (ROB 2) to assess the risk of RCT bias, the quality of the included RCTs was assessed based on the following five domains: randomization process, deviations from intended interventions, missing outcome data, measurement of the outcome, and selection of the reported result. Next, The Risk Of Bias In Non-randomized Studies - of Interventions (ROBINS-I) was used to assess the risk of NRSI bias based on the following seven domains: bias due to confounding, bias in selection of participants into the study, bias in classification of interventions, bias due to deviations from intended interventions, bias due to missing data, bias in measurement of outcomes, bias in selection of the reported result.

### Data Mining Analysis

#### Descriptive Analysis

Frequency statistics of the extracted valid prescriptions were performed using the Microsoft Excel 2019 workbook and included the frequency of point application, frequency of meridian application, frequency of special point application and frequency of positioning distribution.

#### Network Analysis

To generate association rules more effectively and obtain the core acupoint prescriptions of the acupuncture treatments for TDs, the interconnection network of acupoints in all the included prescriptions was analyzed and visually constructed using IBM SPSS Modeler 18.0. Acupoints (defined as the “nodes” of the network) were connected by “lines” in the network. The more frequently used acupoints had thicker connecting lines.

#### Cluster Analysis

First, the acupoints that were used more than 20 times were converted into dichotomous variables, where “1” indicates the occurrence of an acupoint and “0” indicates the absence. Then, cluster analysis was carried out by using “cluster” in IBM SPSS Statistics 26 statistical software. The clustering method was intergroup linking, and the squared Euclidean distance (SED) was used as a measure of relational distance between acupoints to obtain the classification relationship of high-frequency acupoints.

#### Association Rule Mining Analysis

To obtain the high-frequency acupoint pairs, we used the Apriori algorithm of the IBM SPSS Modeler 18.0 software to analyze the association rules. An association rule is expressed in the form A → B. Itemset A represents the “antecedent,” and itemset B represents the “consequent”. The strength of an association rule can be measured by its support, confidence level and lift. The support of A → B refers to the frequency with which itemset A and itemset B appear together in the transactions. The confidence of A → B refers to the conditional probability that itemset A appears in the presence of itemset B. The lift is used to verify that two itemsets are dependent on one another, which makes the rule valuable when the lift's value is larger than 1. We used the support level to determine the probability that A and B occurred simultaneously.

#### Subgroup Analysis

The 5th edition of the Diagnostic and Statistical Manual of Mental Disorders (DSM-5) classifies TD as transient tic disorder (TTD), persistent (chronic) tic disorder or vocal tic disorder (CTD), Tourette syndrome (TS), and other specific or unspecific tic disorders. Only one type of motor tic or vocal tic in TTD and CTD occurs during the disease course. A disease course of <1 year is considered TTD and that of more than 1 year is considered CTD. TS is defined by the presence of both motor and vocal tics. According to the disease course and specific diagnosis of the subjects included in the literature, we performed subgroup analyses of acupuncture prescriptions based on the specific classified diagnosis to further discuss the selection of acupoints for a classified diagnosis.

## Results

### Eligible Studies

**Figure 1:**
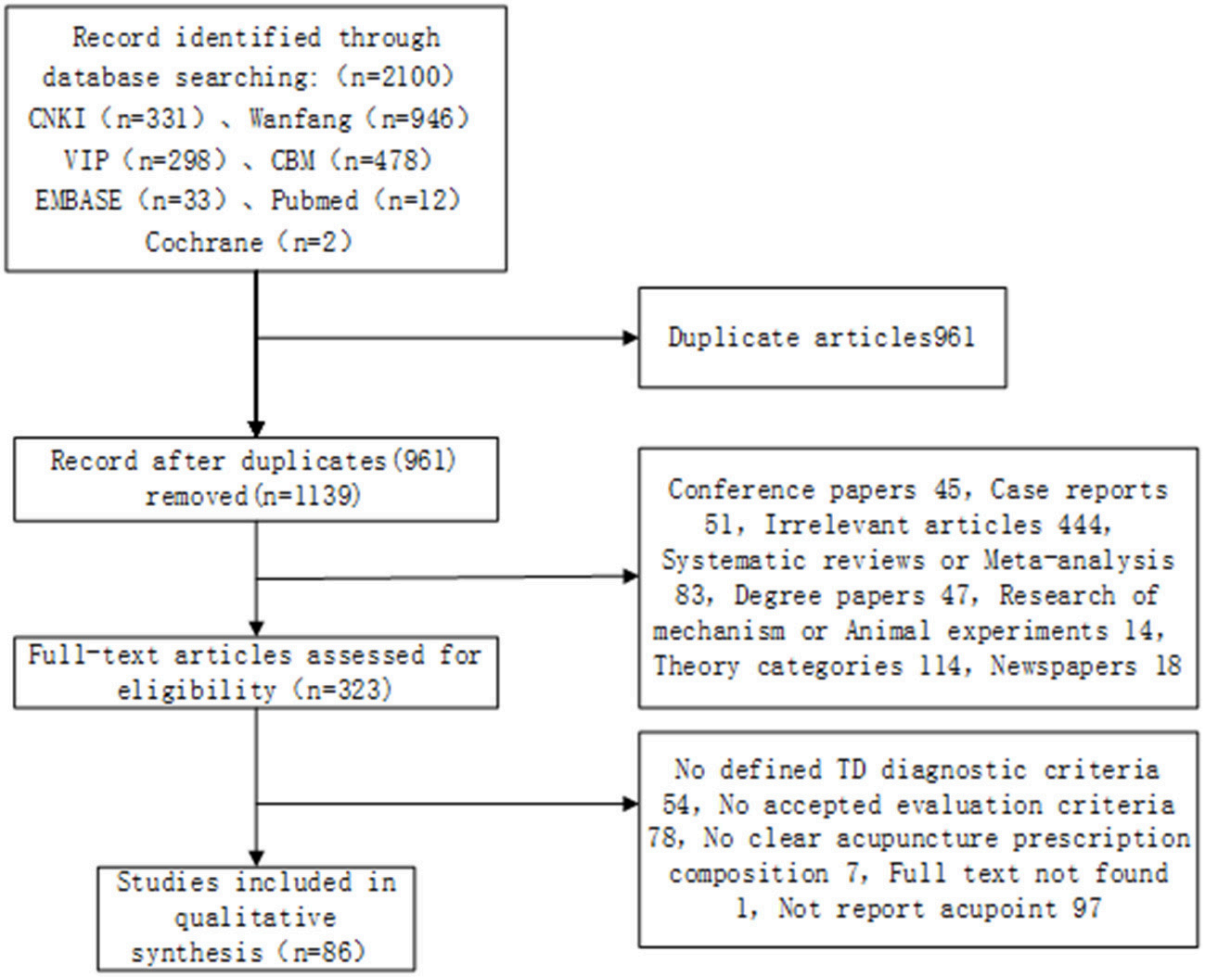
PRISMA flow diagram.

**Figure 2:**
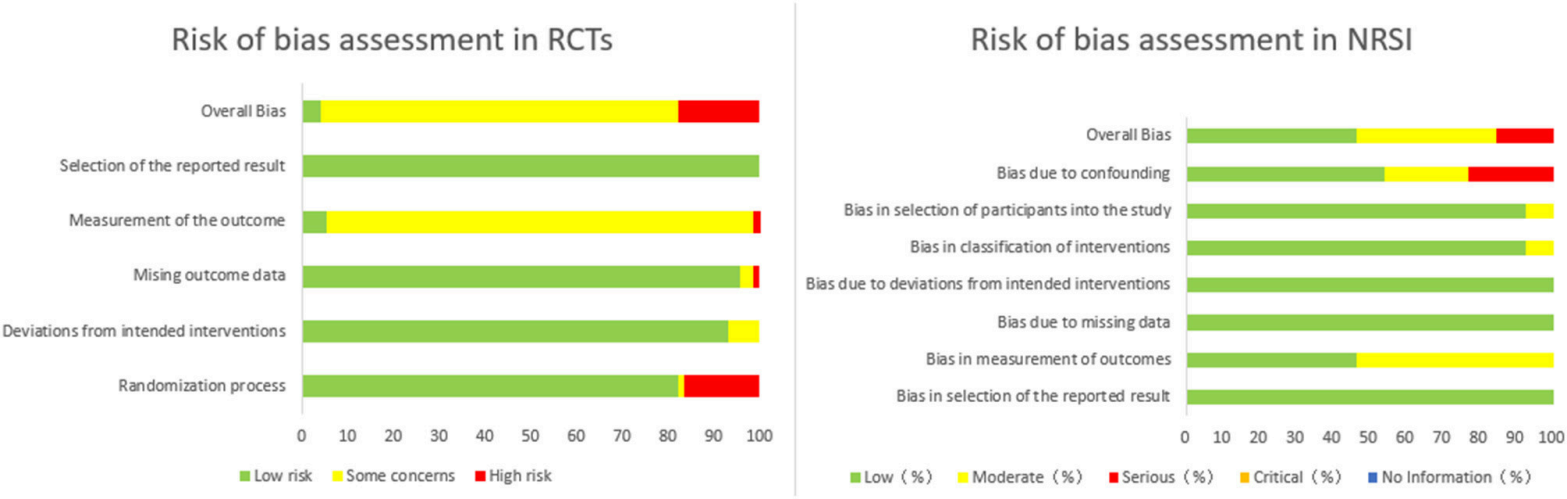
Risk of bias assessment.

The research process is shown in Figure 1 according to the search results. We retrieved a total of 2,100 related articles and removed 961 duplicated studies. Then, we extracted 323 eligible studies by screening the titles and abstracts of the remaining 1,139 studies. Finally, a total of 86 trials were identified based on the inclusion criteria. In all included studies, including 73 RCTs and 13 NRSIs, the overall quality of bias of the RCTs was assessed as low risk (4.1%), some concerns (78.1%), and high risk (17.8%). Because of insufficient allocation concealment and missing outcome data, 94.6% of all RCT studies were rated as high or moderate risk of bias in outcome measures, and 17.8% were rated as high or moderate risk of bias due to randomization process. The overall bias quality of NRSIs was rated as low risk (46.1%), moderate risk (38.5%), and serious risk (15.4%). Higher risks of bias were confounding bias and measurement bias at 46.2 and 53.9%, respectively. The results are shown in Figure 2.

### Frequency of Acupoint Analysis

**Figure 3:**
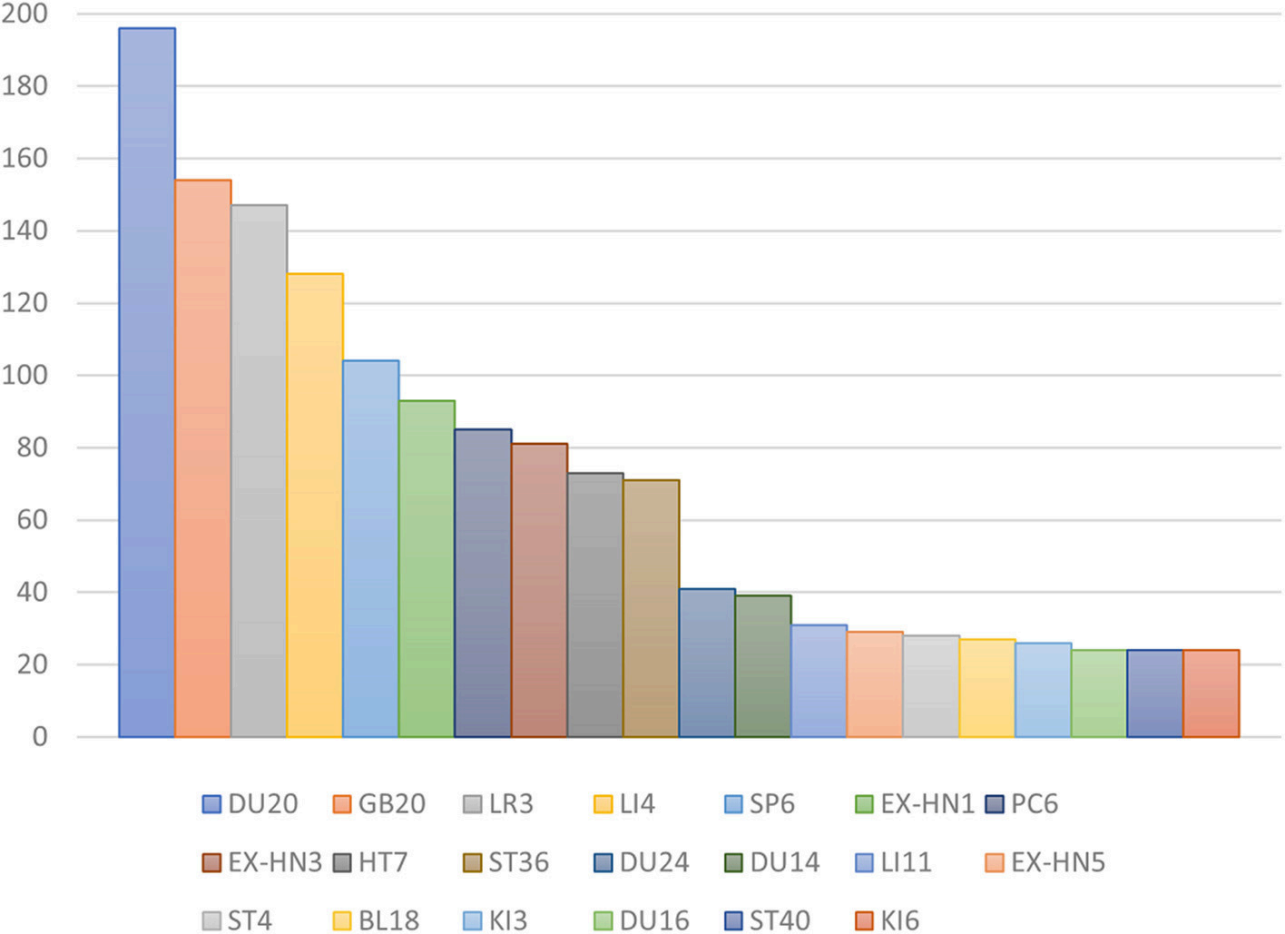
Frequency of acupoint application for TD treatment.

In all, 257 prescriptions were identified involving 121 acupoints with a total frequency of 2,032; 107 of these were traditional acupoints, 11 were extra points, and 3 were the experience acupoint that was not part of any traditional meridian. The five most commonly used acupoints were Bai-hui (DU20), Feng-chi (GB20), Tai-chong (LR3), He-gu (LI4), and San-yin-jiao (SP6) (Table 1; Figure 3). Bai-hui (DU20) was the most frequently used, with a frequency of 76.26% in 257 acupoint prescriptions.

**Table 1:** Frequency of acupoint application for TD treatment.

**NO**.	**Acupoint**	**Frequency**		**Meridian**	**Site of the point**	**Specific points**
1	DU20	196	9.65%	Du meridian	Points of head and neck	Crossing point
2	GB20	154	7.58%	Gallbladder meridian	Points of head and neck	Crossing point
3	LR3	147	7.23%	Liver meridian	Points of lower extremities	Five Shu points, yuan-primary point
4	LI4	128	6.30%	Large intestine meridian	Points of upper extremities	Yuan-primary point
5	SP6	104	5.12%	Spleen meridian	Points of lower extremities	Crossing point
6	EX-HN1	93	4.58%	Extra points	Points of head and neck	
7	PC6	85	4.18%	Pericardium meridian	Points of upper extremities	Luo-connecting point, eight confluent point, crossing point
8	EX-HN3	81	3.99%	Extra points	Points of head and neck	
9	HT7	73	3.59%	Heart meridian	Points of upper extremities	Five Shu points, yuan-primary point
10	ST36	71	3.49%	Stomach meridian	Points of lower extremities	Five Shu points, lower He-Sea point
11	DU24	41	2.02%	Du meridian	Points of head and neck	Crossing point
12	DU14	39	1.92%	Du meridian	Points of head and neck	Crossing point
13	LI11	31	1.53%	Large intestine meridian	Points of upper extremities	Five Shu points
14	EX-HN5	29	1.43%	Extra points	Points of head and neck	
15	ST4	28	1.38%	Stomach meridian	Points of head and neck	Crossing point
16	BL18	27	1.33%	Bladder meridian	Points of back	Back Shu point
17	KI3	26	1.28%	Kidney meridian	Points of lower extremities	Five Shu points, Yuan-primary point
18	DU16	24	1.18%	Du meridian	Points of head and neck	Crossing point
19	ST40	24	1.18%	Stomach meridian	Points of lower extremities	Luo-connecting point
20	KI6	24	1.18%	Kidney meridian	Points of lower extremities	Eight confluent point, crossing point

### Frequency of Meridian Analysis

**Figure 4:**
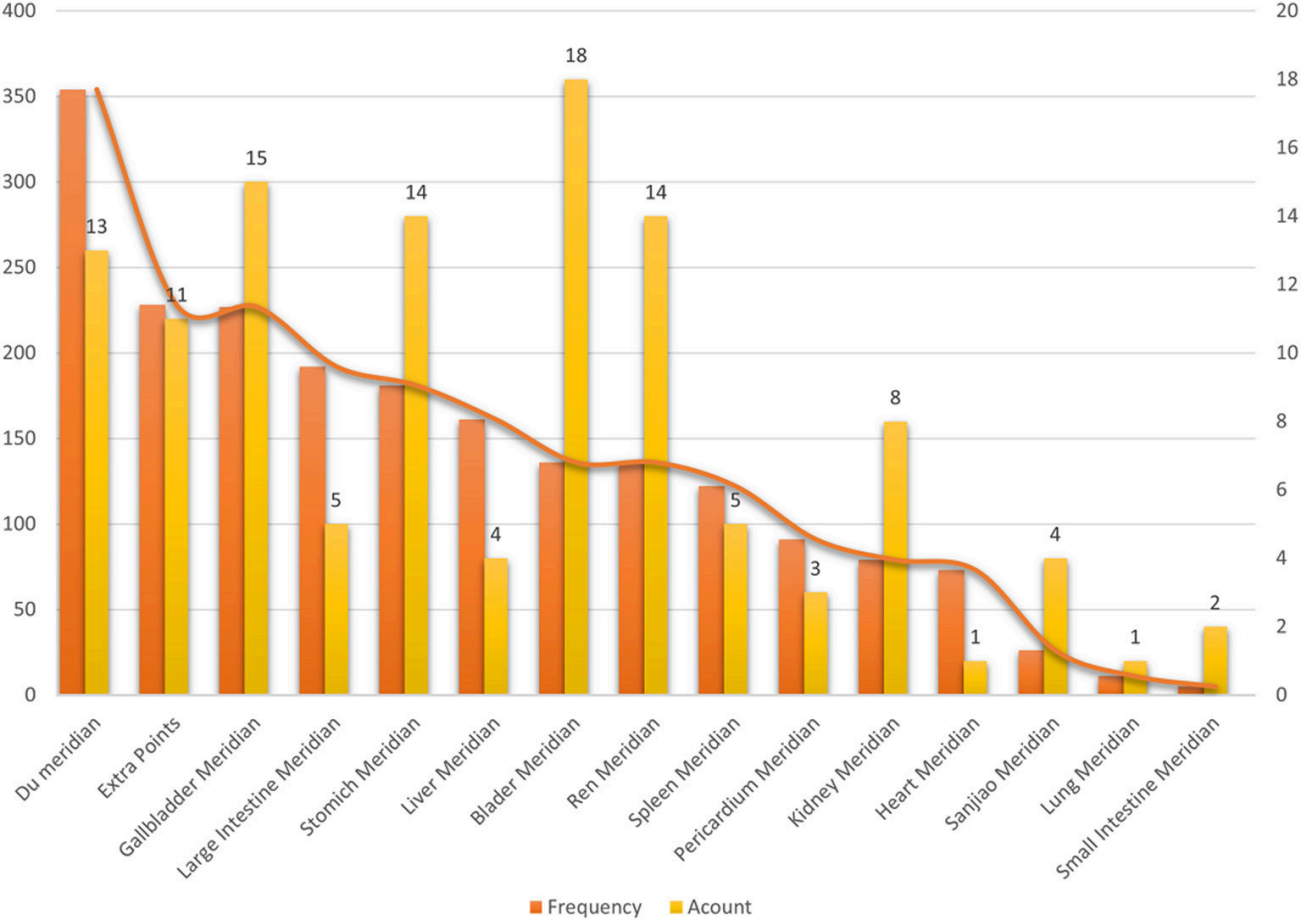
Frequency of meridian application for TD treatment.

Of the 257 acupoint prescriptions, 107 acupoints from the fourteen meridians were used in the treatment of TDs, with a total frequency of 1,794 (Table 2; Figure 4). The distribution detailed in the analysis showed that the Governor Vessel, including 13 acupoints, was the most frequently used at 354 times. The second most used meridian was the gallbladder meridian, which was used 227 times and involved 15 acupoints. The third most used meridian was the large intestine meridian, which was used 192 times and involved five acupoints. The meridian with the most acupoints involved was the bladder meridian, comprising 18 acupoints, but this meridian had a low frequency of use.

**Table 2:** Frequency of meridian application for TD treatment.

**Meridian**	**Frequency**		**Amount**		**Acupoints**
Du meridian	354	17.42%	13	11.02%	DU20 196, DU24 41, DU14 39, DU16 24, DU26 15, DU8 13, DU23 12, DU11 3, DU25 3, DU22 3, DU12 3, DU17 1, DU21 1
Extra points	228	11.22%	11	9.32%	EX-HN1 93, EX-HN3 81, EX-HN5 29, EX-B2 8, EX-HN15 3, EX-HN12 4, EX-HN4 4, EX-HN13 3, EX-B1 1, EX-UE8 1, EX-UE11 1
Gallbladder meridian	227	11.17%	15	12.71%	GB20 154, GB13 13, GB34 11, GB26 8, GB14 8, GB7 6, GB39 6, GB21 5, GB6 5, GB15 3, GB8 2, GB16 2, GB1 2, GB19 1, GB17 1
Large intestine meridian	192	9.45%	5	4.24%	LI4 128, LI11 31, LI20 20, LI15 9, LI10 4
Stomach meridian	181	8.91%	14	11.86%	ST36 71, ST4 28, ST40 24, ST6 17, ST25 11, ST21 8, ST2 5, ST8 5, ST9 4, ST7 4, ST5 1, ST32 1, ST24 1, ST23 1
Liver meridian	161	7.92%	4	3.39%	LR3 147, LR13 8, LR14 4, LR2 2
Bladder meridian	136	6.69%	18	15.25%	BL18 27, BL20 23, BL23 17, BL12 12, BL10 11, BL15 10, BL62 9, BL2 7, BL13 6, BL17 2, BL4 2, BL21 2, BL19 1, BL58 1, BL1 1, BL60 1, BL49 1, BL52 1
Ren meridian	136	6.69%	14	11.86%	RN12 24, RN23 22, RN22 13, RN6 11, RN4 10, RN11 8, RN21 8, RN24 7, RN20 7, RN14 7, RN13 7, RN9 7, RN17 3, RN8 2
Spleen meridian	122	6.00%	5	4.24%	SP6 104, SP1 11, SP9 4, SP10 2, SP4 1
Pericardium meridian	91	4.48%	3	2.54%	PC6 85, PC8 4, PC7 2
Kidney meridian	79	3.89%	8	6.78%	KI3 26, KI6 24, KI18 8, KI21 7, KI26 7, KI1 3, KI4 2, KI7 2
Heart meridian	73	3.59%	1	0.85%	HT7 73
Sanjiao meridian	26	1.28%	4	3.39%	SJ23 11, SJ5 8, SJ17 4, SJ14 3
Lung meridian	11	0.54%	1	0.85%	LU7 11
Small intestine meridian	5	0.25%	2	1.69%	SI18 4, SI9 1

### Specific Acupoint Analysis

**Figure 5:**
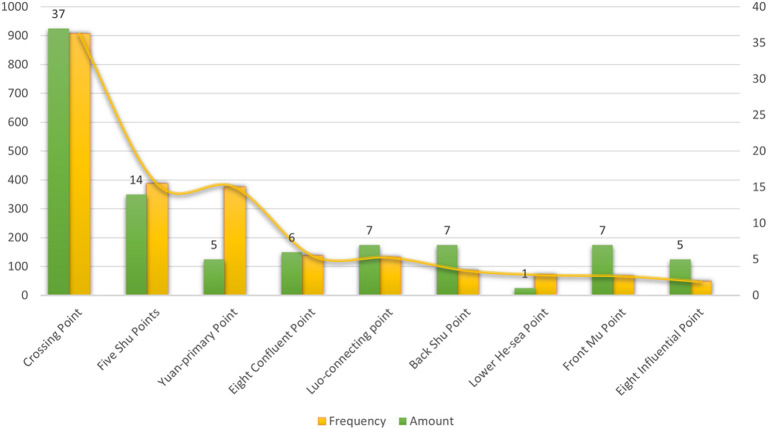
Frequency of special point application for TD treatment.

A total of 67 acupoints were specific acupoints, 17 of which contained multiple attributes. For example, Tai-chong (LR3) is both a Shu point and a Yuan-primary point, and Nei-guan (PC6) is both a Luo-connecting acupoint and an eight confluent acupoint. The top five acupoints were Bai-hui (DU20), Feng-chi (GB20), Tai-chong (LR3), He-gu (LI4), and San-yin-jiao (SP6). The frequency of the crossing acupoints, including 37 acupoints, was far higher than that of the others. The second most used special point was five Shu points, which was used 387 times and involved 14 acupoints (Table 3; Figure 5).

**Table 3:** Frequency of special point application for TD treatment.

**Special point**	**Frequency**	**Amount**	**Acupoints**
Crossing point	907	37	DU20 196, GB20 154, SP6 104, PC6 85, DU24 41, DU14 39, ST4 28, DU16 24, KI6 24, RN12 24, RN23 22, LI20 20, DU26 15, GB13 13, RN22 13, LU7 11, LI15 9, BL62 9, GB14 8, SJ5 8, LR13 8, RN24 7, RN13 7, GB7 6, GB21 5, ST8 5, GB6 5, SI18 4, LR14 4, GB15 3, GB8 2, GB16 2, GB1 2, BL1 3, DU17 1, GB19 1, SP4 1
Five Shu points	387	14	LR3 147, HT7 73, ST36 71, LI11 31, KI3 26, GB34 11, SP1 11, SP9 4, PC8 4, KI1 3, PC7 2, KI7 2, LR2 2, BL60 1
Yuan-primary point	376	5	LR3 147, LI4 128, HT7 73, KI3 26, PC7 2
Eight confluent point	138	6	PC6 85, KI6 24, LU7 11, BL62 9, SJ5 8, SP4 1
Luo-connecting point	132	7	PC6 85, ST40 24, LU7 11, SJ5 8, KI4 2, BL58 1, SP4 1
Back Shu point	86	7	BL18 27, BL20 23, BL23 17, BL15 10, BL13 6, BL21 2, BL19 1
Lower He-Sea point	71	1	ST36 71
Front Mu point	67	7	RN12 24, ST25 11, RN4 10, LR13 8, RN14 7, LR14 4, RN17 3
Eight influential point	48	5	RN12 24, GB34 11, LR13 8, RN17 3, BL17 2

### Distribution of Acupoint Analysis

**Figure 6:**
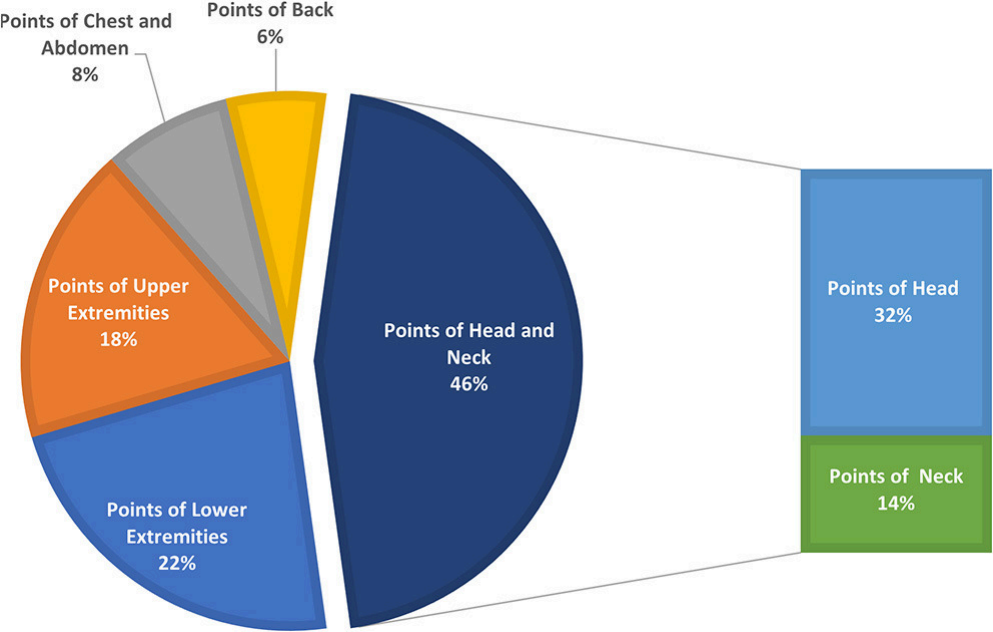
Frequency of site of point application for TD treatment.

The distribution of TD acupoints in acupuncture treatment is detailed in Table 4 and Figure 6. The analysis showed that the acupoints of the head and neck were the main selected acupoints, and the frequency of use accounted for 45.62% of the total.

**Table 4:** Frequency of site of point application for TD treatment.

**No**.	**Site of points**	**Frequency**		**Amount**		**Acupoints**
1	Points of head and neck	927	45.62%	49	40.50%	DU20 196, GB20 154, EX-HN1 93, EX-HN3 81, DU24 41, DU14 39, EX-HN5 29, ST4 28, DU16 24, RN23 22, LI20 20, ST6 17, DU26 15, GB13 13, RN22 13, DU23 12, BL10 11, SJ23 11, EX-B2 8, GB14 8, RN24 7, BL2 7, GB7 6, ST2 5, ST8 5, GB6 5, EX-HN15 3, EX-HN12 4, SI18 4, ST9 4, ST7 4, EX-HN4 4, SJ17 4, DU25 3, GB15 3, DU22 3, EX-HN13 3, GB8 2, GB16 2, BL4 2, GB1 2, An-mian 1, ST5 1, BL1 1, DU17 1, GB19 1, Qian-zheng 1, DU21 1, GB17 1
2	Points of lower extremities	460	22.64%	21	17.36%	LR3 147, SP6 104, ST36 71, KI3 26, ST40 24, KI6 24, GB34 11, SP1 11, BL62 9, Lan-men 8, GB39 6, SP9 4, KI1 3, KI4 2, KI7 2, LR2 2, SP10 2, BL58 1, ST32 1, SP4 1, BL60 1
3	Points of upper extremities	366	18.01%	15	12.40%	LI4 128, PC6 85, HT7 73, LI11 31, LU7 11, LI15 9, SJ5 8, GB21 5, LI10 4, SJ14 3, PC8 4, PC7 2, SI9 1, EX-UE8 1, EX-UE11 1
4	Points of chest and abdomen	157	7.73%	21	17.36%	RN12 24, RN6 11, ST25 11, RN4 10, GB26 8, RN11 8, ST21 8, KI18 8, RN21 8, LR13 8, RN20 7, RN14 7, RN13 7, RN9 7, KI21 7, KI26 7, LR14 4, RN17 3, RN8 2, ST24 1, ST23 1
5	Points of back	122	6.00%	15	12.40%	BL18 27, BL20 23, BL23 17, DU8 13, BL12 12, BL15 10, BL13 6, DU11 3, DU12 3, BL17 2, BL21 2, BL19 1, EX-B1 1, BL49 1, BL52 1

### Cluster Analysis

**Figure 7:**
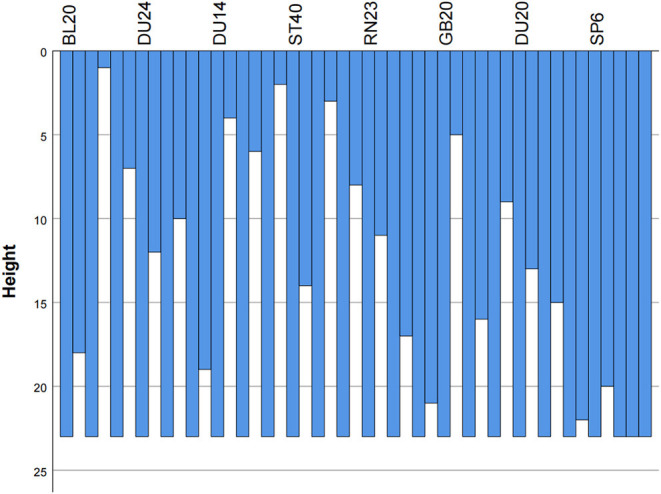
Icicle chart of the cluster analysis of acupuncture for TDs.

**Figure 8:**
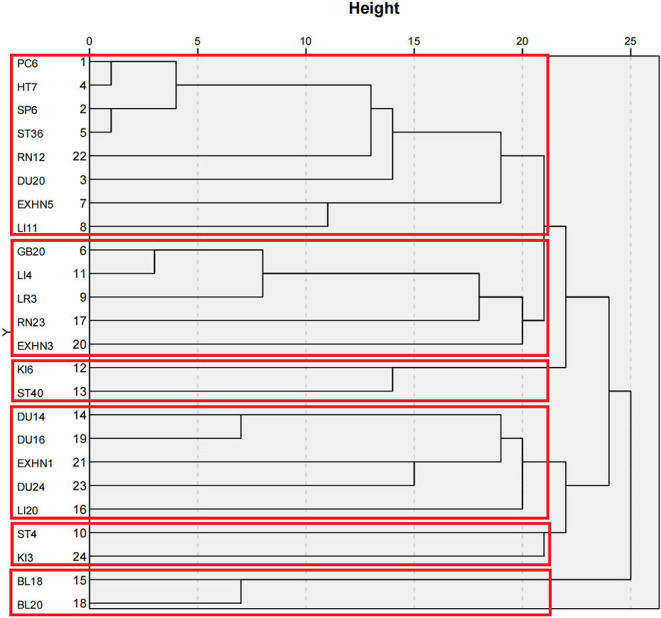
Tree chart of the cluster analysis of acupuncture for TDs.

Cluster analysis was performed on acupoints with frequencies of more than 20 times, resulting in an icicle chart (Figure 7) and tree charts (Figure 8) by using IBM SPSS Statistics 26 statistical software. Cluster analysis is a technique for classifying all subjects into the best homogeneous group based on a measure of similarity. This technique allows us to derive treatment directions and principles for the treatment of TD with acupuncture. By using a clustering algorithm with a distance scale of 15, 24 acupuncture points with frequencies >20 were clustered into a total of six major clusters as follows: Cluster 1, Nei-guan (PC6), Shen-men (HT7), San-yin-jiao (SP6), Zu-san-li (ST36), Zhong-wan (RN12), Bai-hui (DU20), Tai-yang (EX-HN5), and Qu-chi (LI11); Cluster 2, Feng-chi (GB20), Hei-gu (LI4), Tai-chong (LR3), Lian-quan (RN23), and Yin-tang (EX-HN3); Cluster 3, Zhaohai- (KI6) and Feng-long (ST40); Cluster 4, Da-zhui (DU14), Feng-fu (DU16), Si-shen-cong (EX-HN1), Shen-ting (DU24), and Ying-xiang (LI20); Cluster 5, Di-cang (ST4) and Tai-xi (KI3); and Cluster 6, Gan-shu (BL18) and Pi-shu (BL20).

### Association Rule Mining Analysis

**Figure 9:**
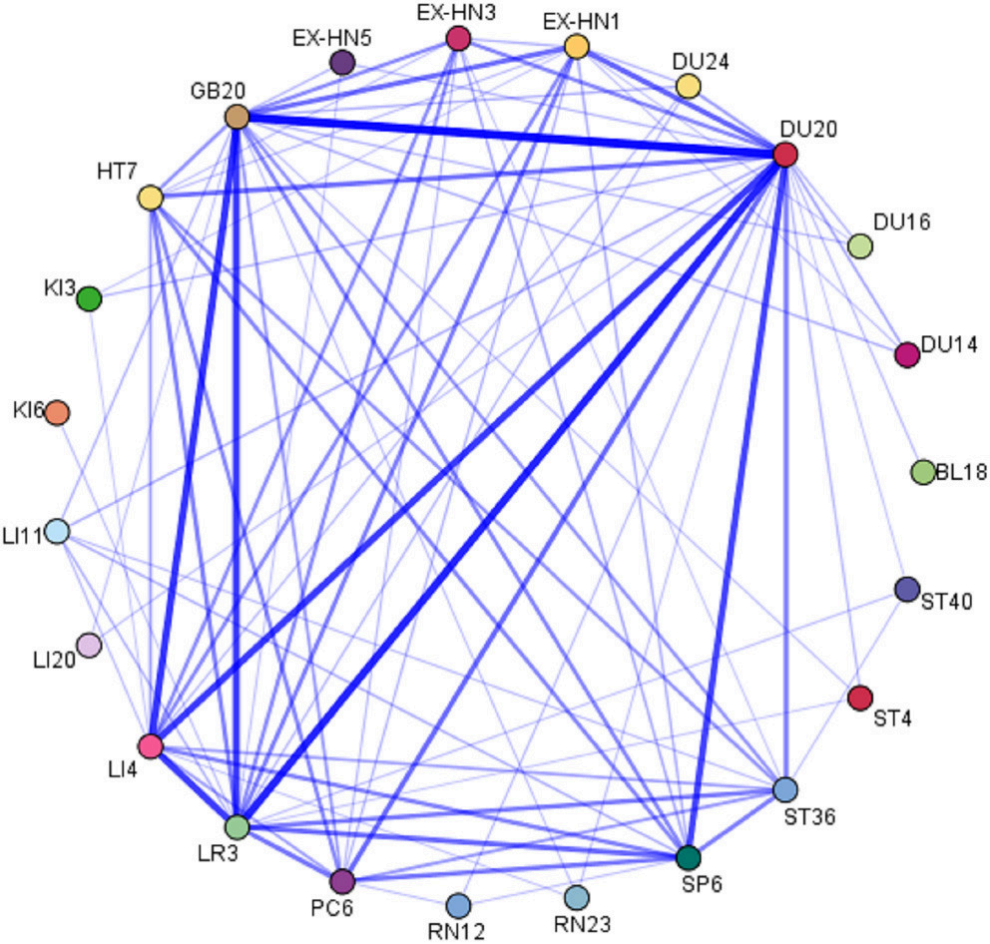
Acupoints association network of acupuncture for TD treatment.

A total of 12 acupoint association rules were obtained using the Apriori algorithm by using IBM SPSS Modeler 18.0, in which the minimum support required was set to 15%, the minimum confidence required was set to 95%, and the maximum number of lift-hand sides was set to 1. In terms of acupoint combinations, the top five combinations with the highest support were {Nei-guan (PC6), San-yin-jiao (SP6)} ≥ {Bai-hui (DU20)}, {Shen-men (HT7), San-yin-jiao (SP6)} ≥ {Bai-hui (DU20)}, {San-yin-jiao (SP6), Feng-chi (GB20)} ≥ {Bai-hui (DU20)}, {Shen-men (HT7), Zu-san-li (ST36)} ≥ {Bai-hui (DU20)}, and {Zu-san-li (ST36), Feng-chi (GB20)} ≥ {Bai-hui (DU20)}. The results are shown in Table 5. In addition, the network diagram drawn from the correlation analysis yielded the core acupoints selected for acupuncture treatment of TDs: Bai-hui (DU20), Si-shen-cong (EX-HN1), Feng-chi (GB20), Nei-guan (PC6), Shen-men (HT7), He-gu (LI4), Zu-san-li (ST36), San-yin-jiao (SP6) and Tai-chong (LR3). The results are shown in Figure 9.

**Table 5:** Association rules of acupoints for TD treatment.

**Post-item**	**Ex-items**	**Confidence**	**Support**	**Lift**
DU20	PC6 and SP6	100.00	25.29	1.32
DU20	HT7 and SP6	100.00	21.01	1.32
DU20	SP6 and GB20	98.15	20.62	1.29
DU20	HT7 and ST36	100.00	17.90	1.32
DU20	HT7 and GB20	97.83	17.51	1.29
DU20	ST36 and GB20	100.00	17.51	1.32
LR3	HT7 and ST36	95.65	17.12	1.70
SP6	ST36 and PC6	100.00	16.73	2.47
DU20	ST36 and PC6	100.00	16.73	1.32
LR3	ST36 and PC6	97.67	16.34	1.73
DU20	PC6 and GB20	100.00	15.56	1.32
DU20	DU24	95.12	15.18	1.25

### Subgroup Analysis

Among the 86 included trials, 5 (5.81%) were clearly divided into TTD (0–1 year) according to the disease course. The remaining trials did not clearly distinguish the treatment according to the disease course. The disease course for the included subjects spanned 0–5 years in 48 (55.81%) and 0–10 years in 17 (19.77%); the remaining 16 (18.60%) did not specify a specific duration of disease. Forty-eight trials (55.81%) investigated TS, and the remaining 33 articles (38.37%) did not make a detailed classified diagnosis or included at least two types of diagnosis.

**Figure 10:**
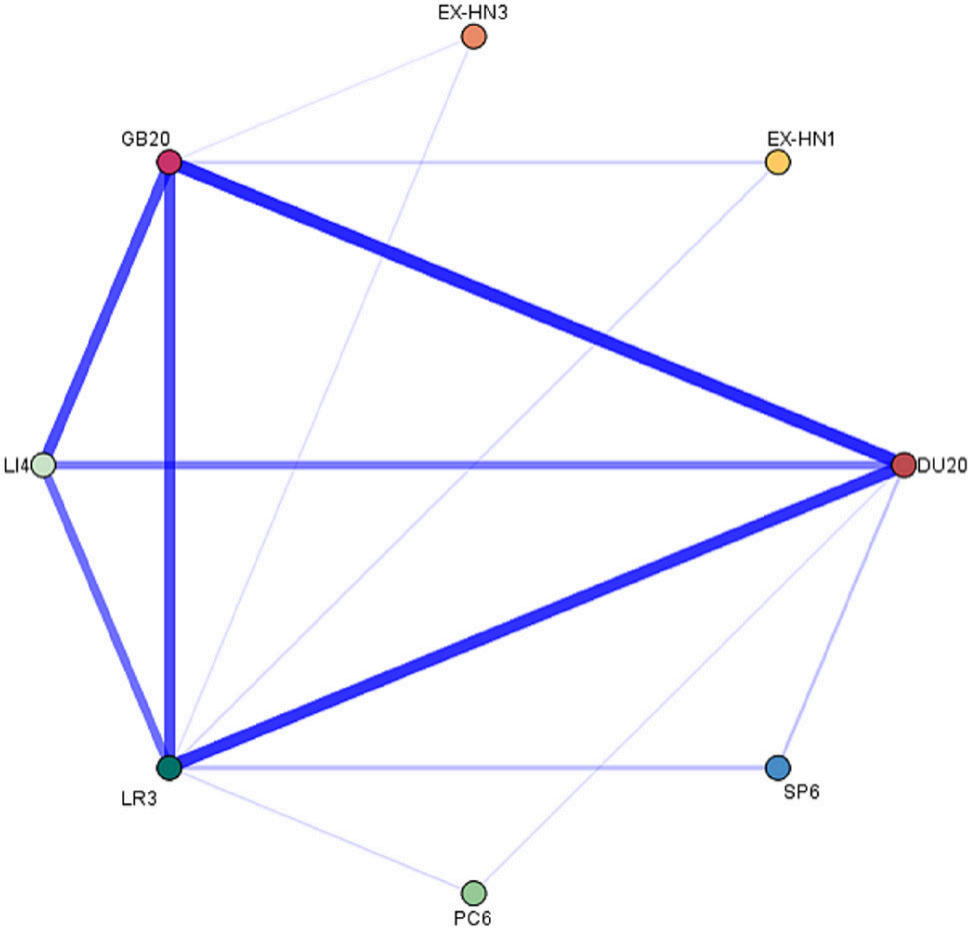
Acupoints association network of acupuncture for TS treatment.

The TS treatment involved 144 prescriptions and 101 acupoints with a total frequency of 1,088; 89 were traditional acupoints, and 12 were extra points. The top five common acupoints were DU20 (111; 10.20%), LR3 (100; 9.19%), GB20 (98; 9.01%), LI4 (79; 7.26%) and EX-HN1 (53; 4.87%). The top 3 common meridians were stomach meridian (14; 13.86%), bladder meridian (14; 13.86%), and gallbladder meridian (13; 12.87%). We set the association rules to Confidence > 90, Support > 20, and Lift > 1, and the acupoint combinations with a high correlation degree were as follows (Figure 10): {Hei-gu (LI4)} ≥ {Bai-hui (DU20)} (Conf 92.41, Supt 50.69, Lift 1.36), {San-yin-jiao (SP6)} ≥ {Tai-chong (LR3)} (Conf 100.00, Supt 33.33, Lift 1.45), and {San-yin-jiao (SP6)} ≥ {Bai-hui (DU20)} (Conf 95.83, Supt 31.94, Lift 1.25).

According to our study, additional acupoints were often used in the TS treatment with tic symptoms in different parts of the body. Forty-two prescriptions were used for facial tics (including frowning, blinking, nose shrugging, pouting, corner of mouth twitching, and drooling), 12 for vocal tics (including roaring and laryngeal ringing), 12 for limb tics, nine for accompanying mental symptoms (including inattention, poor sleep, and irritability), nine for neck tics (including head shaking, shoulder shrugging, and neck twitching), and two for abdominal tics. The acupoints often selected for facial tics were Di-cang (ST4), Tai-yang (EX-HN5), Ying-xiang (LI20), Jia-che (ST6), and Yin-tang (EX-HN3), with Stomach Meridian as the main meridian. When twitching of the limbs occurred, Jian-yu (LI15), Wai-guan (SJ5), and Feng-long (ST40) were often used, with Large Intestine Meridian as the main choice. Lian-quan (RN23) was often used when vocal tics were present. When mental symptoms were also present, Shen-men (HT7) and Nei-guan (PC6) were often selected. Tian-zhu (BL10), Ren-ying (ST9), and Lie-que (LU7) were often selected when neck tics occurred.

## Discussion

The overall quality of the 86 publications in this study was biased toward moderate to high risk, and 68 RCTs (93.2% of all RCTs) were deemed moderate to high risk of bias in outcome measures. The main reason is that acupuncture treatment is different from drug treatment. Additionally, achieving complete double-blindness in RCT studies is challenging. Most types of placebo acupuncture cannot make the experimenter single-blind, and most RCTs use drug control, making it impossible to achieve single blinding of subjects, resulting in a higher risk of bias in outcome measures due to unblinding. A moderate to high risk of bias in the randomization process was found in 17.8% of the studies because they used inappropriate allocation methods, such as allocation according to the order of patient visits, making the allocation concealment insufficient. The low risk percentages for bias in deviations from intended interventions and bias in missing outcome data were 93.1 and 95.8%, respectively. Thus, most of the patients showed good adherence to acupuncture treatment and completion of the treatment course; only a few studies had missing data results due to study withdrawal for reasons such as adverse reactions to control medications.

Only 1 of the 13 NRSIs was a retrospective study, and 92.3% had a low risk of bias in selecting participants. Additionally, all the subjects in the 13 NRSIs showed good compliance, and no omission of clinical data or selective reporting occurred. The overall risk of bias was low and medium, representing a good reference value. However, this study had many limitations. For example, six NRSIs, accounting for 46.2% of all NRSIs, were associated with confounding biases such as traditional Chinese medicine, and all NRSIs were self-control studies before and after. Differences in different individuals and stages of the same individual may have affected the study findings. Therefore, the control of confounding bias in clinical research must be further improved. Additionally, the use of blinding was not clarified in any of the NRSI outcome measures; thus, the bias in the outcome measures was evaluated as medium risk. Some clinical studies had certain flaws in the initial design; thus, the rigor of clinical study protocol design must be improved.

The evaluation of the efficacy of acupuncture clinical trials is closely related to the process of acupuncture treatment, which includes the selection and combination of acupoints, interventions and treatment protocols. The selection and combination of acupuncture points are based on channel diagnostics and the syndrome differentiation of channel theory. Therefore, the use of data mining techniques to analyze the use of acupuncture points in clinical trials of acupuncture helps improve the treatment protocols in clinical and experimental studies of acupuncture for TDs and provides more effective options for acupuncture point selection and combination selection.

This study shows that the treatment of TDs emphasizes the main treatment principles and concepts of coordinating yin and yang, tonifying qi and blood, dispelling pathogenic wind and eliminating phlegm. It focuses on the use of the Bai-hui (DU20) and the Governor Vessel and on the selection of the crossing acupoint and the five Shu points. The choice of the distribution of acupoints focuses on the head and neck. The potential point combinations were Bai-hui (DU20), Nei-guan (PC6), and San-yin-jiao (SP6).

In this study, we used data mining to systematically summarize the prescriptions of acupuncture therapy for TD. The results show that Bai-hui (DU20) was the core acupoint used for TD treatment. Bai-hui (DU20) was one of the main acupoints of 76.26% of all prescriptions assessed in this study. It belongs to the Governor Vessel and is the intersection of the triple energizer meridian, gallbladder meridian, bladder meridian, and liver meridian. Therefore, it is also known as “san yang wu hui”. Bai-hui (DU20) is located on the top of the head. TCM holds the opinion that “all yang channels converge overhead”. Lingshu Jing, an ancient Chinese literature, indicates that all qi channels circulate in the head and enter the brain. According to this theory, acupuncture at Bai-hui (DU20) can mobilize the Qi of the whole body into the brain. Several studies have demonstrated that the onset of TD is associated with decreased dopamine levels due to striatal dopaminergic neuronal hyperactivity or postsynaptic dopamine receptor hypersensitivity (9, 10). In contrast, acupuncture in Bai-hui significantly inhibited the expression of dopamine receptors in the striatum and substantia nigra by modulating the dopamine system in the striatum, substantia nigra, and prefrontal cortex to control tics (11).

Another commonly used point was Feng-chi (GB20), with a total frequency of 154. Feng-chi (GB20) belongs to the bile meridian and is located under the occipital bone, flush with Feng-fu (GU16), between the sternocleidomastoid and oblique muscles, 1 inch into the hairline. Feng-chi (GB20) is anatomically located at the projection of the vertebral artery on the body surface. Stimulation of the neck muscles by acupuncture with Feng-chi (GB20) improves blood supply to the vertebral-base artery system and accelerates blood flow to the brain, achieving the effect of improving blood supply to the brain. It is now believed that there is a correlation between the onset of TD and reduced 5-HT and that acupuncture with Feng-chi (GB20) can increase central 5-HT levels and reduce the release of 5-HT in peripheral blood.

According to TCM theory, the twitching symptom in TD is considered to be tendon injury (12), and tendons are closely related to the liver and involve the remaining four organs. In the Su Wen, it is believed that yang qi can replenish shen qi and nourish tendons and that the Governor Vessel is the master of the yang vessel. Therefore, all muscles along twelve regular channels need to be warmed and pushed through the yang qi of the Governor Vessel to function. In our study, the Governor Vessel was also the most frequently used meridian. It travels along the posterior midline of the human body, borders the Conception Vessel and closely connects to all the zang-fu organs. At the same time, it regulates the yin and yang qi and blood of the whole body. The Great Compendium of Acupuncture and Moxibustion documents that the Governor Vessel crosses the top of the head and enters the brain. Therefore, it can be assumed that the Governor Vessel has a therapeutic effect on brain diseases, especially in patients who also have mental disorders. Therefore, we believe that the use of the Governor Vessel meridian has a therapeutic effect on brain disorders, which has a positive effect on TD patients with concomitant mental disorders.

In addition, the crossing acupoints, including Bai-hui (DU20), Feng-chi (GB20), and San-yin-jiao (SP6), are specific acupoints commonly used in the treatment of TDs, accounting for 44.6% of the total frequency of acupoints. The crossing acupoint is the acupuncture point where the meridians meet, which can take all the main functions of the intersecting meridians. The crossing acupoints selected in this study mostly intersect with the Governor Vessel and the gallbladder meridians. According to this result, it can also be reverified that acupuncture treatment for TDs attaches importance to the Governor Vessel. The top 3 acupoints of the crossing acupoint were Bai-hui (DU20), Feng-chi (GB20), and San-yin-jiao (SP6). We identified these three acupoints as the most commonly used acupoint combinations in TD treatment by using association rule mining techniques. Feng-chi (GB20) belongs to the gallbladder meridian, which intersects with the triple energizer meridian and the Yang Heel Vessel. It is on the posterior side of the head, located in the depression between the bilateral sternocleidomastoid muscles and the trapezius muscles. It treats muscle twitches of the head and improves blood circulation in the brain. San-yin-jiao (SP6) is the place where qi and blood substances meet in the three yin meridians of the foot, which can strengthen the spleen and blood and tonify the liver and kidneys. After the crossing acupoint, the five Shu points have the highest frequency of application. The top 3 acupoints were Tai-chong (LR3), Shen-men (HT7), and the Yuan-Source acupoint of the liver meridian and the heart meridian. The original qi of the internal organs starts from the kidney and is injected into five zang organs and six fu organs through Sanjiao. The Yuan-Source acupoint is the node through which the original qi of the organs passes and remains. Therefore, it can be used to treat the disease of the corresponding organs. TCM considers that liver lesions cause TD, and the heart, spleen and kidneys are also affected. Choosing the corresponding yuan primary points can replenish the qi of the organs.

From the study, acupuncture treatment of TD mainly uses acupoint sites in the head and neck. On the one hand, acupuncture at points of the head will stimulate the corresponding functional areas of the brain, which can improve blood circulation in the brain, improve the oxygen carrying capacity of brain cells, and finally promote the repair of neuronal cells (13). Stimuli of the thalamus and the globus pallidus are effective in controlling twitching episodes (14). Nerve fibers emanating from the thalamus project to the frontal and parietal cortices of the brain. This coincides with the findings of our study. On the other hand, ~58% of TD patients have recurrent neck twitches (15). Repeated hard stretching of the neck in jerks can lead to spinal cord damage. Therefore, TD patients are more likely to suffer from cervical spine disorders than the general population (16). Studies have demonstrated that TD affects the medulla oblongata and has a high potential for subsequent progression to severe cervical spondylosis (17–21). In addition to head acupuncture points, our study indicated that neck acupoints such as Feng-chi (GB20) and Da-zhi (DU14) were also used. We believe that acupuncture of the neck may have a better effect in regulating the action of the medulla oblongata and blocking the progression of TDs.

By using a clustering algorithm, 24 acupoints with a frequency >20 were clustered into six major clusters, signifying the main treatment principles and concepts of coordinating yin and yang, tonifying qi and blood, dispelling pathogenic wind and eliminating phlegm.

The TCM pathogenesis of TDs has been summarized by some scholars (22). TCM distinguishes two general pathogeneses of TD, a “deficiency” pathogenesis and an “excess” pathogenesis. The former appears in TD as an imbalance in qi, blood, yin and yang due to the weakening of the five zang organs and six fu organs. The other one occurs in TDs caused by wind-phlegm, which causes tendon injuries and weakness of yin and yang. The wind-phlegm is twisted and reaches the head and face, flies to the limbs, and migrates to the airway. Thus, TDs manifest as facial muscle twitching, limb twitching, mouth salivation and others. According to the above article, the pathogenesis of TD is mainly the deficiency of the five zang organs, imbalance of yin and yang, and wind-phlegm strangulation, matching the treatment direction we have concluded. Thus, acupoint Cluster 1, Nei-guan (PC6), Shen-men (HT7), San-yin-jiao (SP6), Zu-san-li (ST36), Zhong-wan (RN12), Bai-hui (DU20), Tai-yang (EX-HN5), and Qu-chi (LI11), and Cluster 6, Gan-shu (BL18) and Pi-shu (BL20), were chosen to invigorate the heart, liver and spleen, benefit both qi and blood and achieve the effect of dispelling wind-evil. Cluster 2, Feng-chi (GB20), Hei-gu (LI4), Tai-chong (LR3), Lian-quan (RN23) and Yin-tang (EX-HN3), was used to dispel pathogenic wind for resolving convulsions. Cluster 3, Zhaohai- (KI6) and Feng-long (ST40), and Cluster 5, Di-cang (ST4) and Tai-xi (KI3), were used to invigorate the spleen and kidney and dissipate phlegm. Cluster 4, Da-zhui (DU14), Feng-fu (DU16), Si-shen-cong (EX-HN1), Shen-ting (DU24), and Ying-xiang (LI20), was used to coordinate yin and yang and draw qi and blood into the brain.

Network diagrams derived from association rule mining analysis yielded the core acupuncture points with the strongest associations, which revealed potentially effective acupoint prescriptions for TD treatment; these points were Bai-hui (DU20), Si-shen-cong (EX-HN1), Feng-chi (GB20), Nei-guan (PC6), Shen-men (HT7), He-gu (LI4), Zu-san-li (ST36), San-yin-jiao (SP6), and Tai-chong (LR3) (Figure 8).

We used association rule mining technology to identify the most commonly used combinations of acupuncture points for the treatment of TDs. The core group is Bai-hui (DU20), Nei-guan (PC6), and San-yin-jiao (SP6). As mentioned earlier, TCM considers the deficiency of the five zang organs and the imbalance of yin and yang as the mechanism of TD attacks. Nei-guan (PC6) is the Luo-connecting point of the pericardium meridian, and it is also one of the eight influential acupoints. It is connected to the Yin Link Vessel. It has the function of nourishing blood and resolving stagnation for tranquilization. San-yin-jiao (SP6) belongs to the spleen meridian. It tonifies the spleen and kidneys and the innate deficiency. Experimental studies have shown that acupuncture with Nei-guan (PC6) and Shui-gou (DU26) protects neuronal cells by reducing the abnormally elevated mRNA expression of IL-1RI and TNFR-I in brain tissue (23). Acupuncture with Nei-guan (PC6), Shui-gou (DU26), and San-yin-jiao (SP6) improves the reperfusion function of the brain and reduces the occurrence of neuronal cell apoptosis (24). Therefore, we believe that the combined use of Nei-guan (PC6) and San-yin-jiao (SP6) regulates the flow of qi and blood in the body. When paired with Bai-hui (DU20), they combine yin and yang to harmonize qi and blood. However, a three-acupoint combination was seldom used as the prescription in the treatment of TDs. We therefore conclude that acupoint prescriptions for the treatment of TDs often consist of different combinations of commonly used acupoints on commonly used meridians.

Only five trials explicitly included only participants in the short-term disease course, while others included participants in both the short- and long-term disease course. Participants with a disease course of 0–5 years accounted for 61.63% of the trials, and participants with a disease course >5 years numbered fewer than those with a disease course of <5 years. This finding might be related to the benign prognosis of TD patients who usually have symptoms that gradually lessen after puberty. Although we have not found strong evidence that the earlier is the treatment, the better is the effect, a few trials suggest that acupuncture with a short-erm disease course is more effective than that with a long-term disease course.

The TS trials accounted for 55.81% of all trials. The core acupoint prescription of TS treatment with the strongest correlation included He-gu (LI4), Feng-chi (GB20), Tai-chong (LR3), Bai-hui (DU20), Yin-tang (EX-HN3), Si-shen-cong (EX-HN1), San-yin-jiao (SP6), and Nei-guan (PC6), which is essentially the same as the core acupuncture point prescription for TD treatment. Yin-tang (EX-HN3) is a new core point that is also the most frequently used point when facial tics occur. It is located in the center of the eyebrow and belongs to the Governor Vessel, which is considered by TCM to induce resuscitation. Unlike TD treatment, its core group includes He-gu (LI4) and Feng-chi (GB20). He-gu (LI4) is the Yuan-Source acupoint of the large intestine meridian, where the qi and blood gather. In TCM theory, the large intestine meridian is also considered a meridian rich in qi and blood; therefore, He-gu (LI4) significantly affects the body's regulation of qi and blood throughout the body and its combination with Feng-chi (GB20) is ideal.

As mentioned previously, the clinical picture of TS is complex and varied, with both motor and vocal tics occurring during the disease course. TS treatment was often used with specific additional acupoints at different local sites where tics occur. For example, the most commonly used additional acupoint for facial tics is Di-cang (ST4). Acupuncture Di-cang (ST4) raises precentral gyrus and postcentral gyrus signals (25) and may achieve the effect of controlling the motor sensation of the face. Lian-quan (RN23) is often used for vocal tics. It is located between the thyroid cartilage and hyoid bone. Its deep part comprises branches of the sublingual nerve and swallowing nerve. Studies have reported that acupuncture with Lian-quan (RN23) stimulates the laryngeal muscles (26), which may control vocal tics to some extent. The acupoint in the human being has two functional states—sensitization state and rest state. When the human body has disease, the acupoints on the surface of the body will be sensitized. When the sensitized acupoints are stimulated externally, they trigger larger internal stimulation (27) and may reflect the significance of local acupuncture at the location where the tics occur. In summary, TS treatment is often performed with proximal points in the selection of additional acupuncture points.

## Limitations

The present study has several limitations that need to be considered. First, the methodological quality of the included studies was relatively low. This is because it is extremely difficult to achieve complete double-blindness for acupuncture treatment and sufficient concealment of treatment allocation, resulting in incomplete outcome data. Some studies utilized treatment groups that combined other treatments, such as head and hand acupuncture, all of which led to potentially biased efficacy results. More rigorous clinical studies of acupuncture for TD need to be developed in the future to improve the quality of evidence. Second, all trials included participants with at least two levels of severity and did not delineate these levels more carefully, and most trials did not make a careful distinction in the selection of the timing of the intervention. Thus, further studies are required. Third, the more common criteria to evaluate the efficacy of this disease are mainly based on rating scales such as the YGTSS score, which are subjective, possibly leading to biased results. More objective indicators of acupuncture for TD will be needed in the future to enhance the quality of the evidence. Finally, the potential prescriptions extracted through data mining are the result of data integration. Further animal experiments and clinical trials are still needed to verify whether these prescriptions are practical and feasible.

## Conclusion

In summary, the current study investigated the potential acupoints and combinations for TD treatment based on a data mining analysis of published studies. Bai-hui (DU20), Feng-chi (GB20), Tai-chong (LR3), He-gu (LI4), and San-yin-jiao (SP6) appeared to be the most frequently used acupoints for TD treatment. The Governor Vessel was the more commonly selected meridian, which demonstrates a significant role in the treatment of TD. Additionally, the selection of the crossing acupoint and the five Shu points was found to be important. The selection of acupuncture points focused on the head. Cluster analysis indicated the treatment principle of harmonizing yin and yang, tonifying qi and blood, dispelling wind and resolving phlegm. The potentially effective acupoint prescriptions were revealed by a network analysis. There were Bai-hui (DU20), Si-shen-cong (EX-HN1), Feng-chi (GB20), Nei-guan (PC6), Shen-men (HT7), He-gu (LI4), Zu-san-li (ST36), San-yin-jiao (SP6), and Tai-chong (LR3). Association rule mining demonstrated that the combination of Bai-hui (DU20) + Nei-guan (PC6) and San-yin-jiao (SP6) was a potential acupoint combination that should be selected with priority in TD treatment. The core acupoint prescription of TS treatment comprised He-gu (LI4), Feng-chi (GB20), Tai-chong (LR3), Bai-hui (DU20), Yin-tang (EX-HN3), Si-shen-cong (EX-HN1), San-yin-jiao (SP6), and Nei-guan (PC6). The core group comprised He-gu (LI4) and Feng-chi (GB20). Additionally, the treatment is often performed with proximal points in the selection of additional acupuncture points. Overall, this study provides valuable information for the selection of acupoints for the clinical acupuncture treatment of TDs.

## Author's Note

Data mining is a technology that has emerged in recent years with the development of the Web, artificial intelligence, and database technology. It is used to conditionally filter out valid and credible information from abundant random source data and derive regular and potential information after advanced processing to achieve efficient learning. Tic disorders are neuropsychiatric disorders occurring primarily in children and adolescents. They can cause physical and psychological harm to the patient. The relationship between the efficacy of the clinical acupuncture treatment of tic disorders and the acupuncture treatment process and the selection and combination of acupoints based on meridian and acupoint theory must be evaluated. The volume of literature on acupuncture for treating tic disorders has been increasing recently. Using data mining techniques to analyze the use of acupuncture points in the clinical treatment of tic disorders and to explore the potential patterns may enable the selection of more effective acupuncture points and combination protocols for the clinical acupuncture treatment of tic disorders and provide new therapeutic ideas.

## Data Availability Statement

The original contributions presented in the study are included in the article/supplementary material, further inquiries can be directed to the corresponding authors.

## Author Contributions

JC analyzed and visualized the data and wrote the original manuscript. YX conceived and designed the study protocol and wrote the original manuscript. QL was involved in the visualization of the data. QL, WC, and YW searched the articles and screened the eligible literature. YC and ZG extracted the data. JZ was involved in the revision of the manuscript. ZQ and JF were responsible for the review process and participated in the critical revision of the manuscript. All the authors read and approved the final manuscript.

## Funding

This study was funded by the Chinese Medicine Specialty Project of the Department of Acupuncture and Moxibustion, Shenzhen Hospital of Guangzhou University of Chinese Medicine (Chinese Medicine Division of Shenzhen Municipal Health Commission, 2019, No: 22).

## Conflict of Interest

The authors declare that the research was conducted in the absence of any commercial or financial relationships that could be construed as a potential conflict of interest.

## Publisher's Note

All claims expressed in this article are solely those of the authors and do not necessarily represent those of their affiliated organizations, or those of the publisher, the editors and the reviewers. Any product that may be evaluated in this article, or claim that may be made by its manufacturer, is not guaranteed or endorsed by the publisher.
